# Preservation of Antioxidant and Immunomodulatory Properties in a Heat-Treated Probiotic Blend: Insights from Preclinical Models

**DOI:** 10.3390/microorganisms14081820

**Published:** 2026-08-18

**Authors:** Daniel González-Hedström, Silvia Llopis, Nuria González, Ester Pardo, Verónica Navarro, Jennifer Redondo, Guillermo García-Lainez, Valerio Rossini, Miren Maicas, Verónica Martínez-Ríos, Empar Chenoll, Patricia Martorell

**Affiliations:** Archer Daniels Midland, Nutrition, R&D Health & Wellness, Biopolis S.L. Parc Cientific Universitat de València, C/Catedrático Agustín Escardino Benlloch, 9, 46980 Paterna, Spain

**Keywords:** oxidative stress, inflammation, *Caenorhabditis elegans*, leaky gut, AMPK, sports nutrition, energy, ATP, mitochondria, heat-treated

## Abstract

In a previous clinical trial, it was shown that a probiotic blend (*Bifidobacterium longum* CECT 7347 (Esflorin1™), *Lacticaseibacillus rhamnosus* CECT 8361 (BPL15) and *Lacticaseibacillus casei* CECT 9104 (BPL4)) reduced oxidative stress in males engaging in intense exercise. The present study evaluated whether the probiotic blend’s functional properties are preserved after heat treatment and investigated the underlying mechanism of action using preclinical models. The antioxidant, immunomodulatory and intestinal effects of the heat-treated and probiotic blends were assed using in vitro assays and *Caenorhabditis elegans* (*C. elegans*) models. The heat-treated blend preserved the antioxidant activity by scavenging free radicals, reducing intracellular reactive oxygen species and enhancing survival in *C. elegans* under oxidative stress, possibly via sod-3 upregulation in the CF1553 strain. It also enhanced mitochondrial biogenesis and ATP production in C2C12 via AMPK phosphorylation. Both versions attenuated a gut inflammatory response, improved intestinal barrier in Caco-2 cells and *C. elegans* and exhibited immunomodulatory activity in U937 macrophages at the tested concentrations. These findings support the heat-treated blend as a promising postbiotic supplement to manage oxidative stress and other physiological alterations associated with intense exercise.

## 1. Introduction

Moderate physical activity has a beneficial and multidirectional effect on the human body, causing physiological and metabolic adaptations. Among others, it increases mitochondrial biogenesis and function, metabolic enzyme activity and glycogen storage in skeletal muscle [1], in addition to favoring protein synthesis depending on the intensity of training [2]. However, intense or unaccustomed exercise can increase oxidative stress, inflammation and intestinal permeability, while suppressing the immune system. These adverse effects depend on the duration and intensity of the exercise, with athletes and highly active individuals at greater risk [3,4].

Oxidative stress can be defined as the disturbance in the balance between the production of reactive oxygen species (ROS) and antioxidant defenses. Hydrogen peroxide, superoxide anion and hydroxyl radicals are the most common ROS generated in biological systems as metabolic byproducts. These react with biomolecules such as lipids or proteins, giving rise to nitrosylated or oxidized products, which may have a detrimental effect on cellular functions [5]. Moderate levels of ROS are necessary for the production of normal muscle force and the enhancement of muscle antioxidant capacity [6], but excessive ROS released as byproducts of cellular respiration by mitochondria can lead to muscle fatigue and contractile dysfunction [4]. The tissue damage produced by excessive training and by ROS results in a local acute inflammatory response, increasing the levels of pro-inflammatory cytokines such as interleukin (IL)-1β, tumor necrosis factor α (TNF-α), IL-6 and IL-8 [7,8]. Among the different antioxidant defense mechanisms that the body has developed are the antioxidant enzymes, with super oxide dismutase (SOD), catalase (CAT), glutathione reductase (GR) and glutathione peroxidase (GPx) being the most prominent. The expression of these enzymes is primarily regulated through the nuclear factor erythroid 2-related factor 2 (Nrf2) signaling pathway [9]. Additionally, the AMP-dependent protein kinase (AMPK), a key regulator of energy metabolism, plays a critical role in antioxidant enzyme regulation during exercise and dietary interventions [10]. Exercise or energy stress can activate the AMPK pathway in skeletal muscle, as muscle contraction elevates intracellular adenosine monophosphate/adenosine triphosphate (AMP/ATP) and adenosine diphosphate/adenosine triphosphate (ADP/ATP) ratios [11,12]. Once activated, the AMPK promotes substrate uptake, oxidative metabolism, mitochondrial biogenesis and intracellular ATP production [12,13].

Beyond the effects on the muscle, excessive training loads may induce intestinal dysbiosis, resulting in mucosal dysfunction and increased intestinal permeability [14]. The elevated intestinal permeability may facilitate translocation of pathogens and toxins into the bloodstream, increasing susceptibility to infections and triggering local and systemic inflammatory pathways through the induction of IL-1, TNF-α and interferon gamma (IFN-γ) secretion [15]. In fact, several studies have observed higher resting values of IL-1β, IL-4 and IL-8 cytokine in master athletes compared to young or middle-aged adults [16,17]. Moreover, prolonged, high-intensity exercise is often linked to post-exercise immunosuppression and increased infection risk due to cytokine imbalance [18].

Therefore, several studies have evaluated different nutritional interventions to counteract exercise-induced side effects [19,20]. According to the International Scientific Association for Probiotics and Prebiotics (ISAPP), probiotics are “*live microorganisms that, when administered in adequate amounts, confer a health benefit on the host*” [21]. In this context, a position stand from the International Society of Sports Nutrition (ISSN) highlighted areas where probiotics may work to aid athletes in meeting the demands of training and competition [22]. The areas include immune modulation to avoid or minimize the impact of illness and infection and to improve or enhance the ability to maintain healthy gut permeability. Beyond those areas, probiotic interventions have been shown to reduce lipid-related or DNA-related oxidative stress biomarkers, increase plasma levels of antioxidants and reduce inflammation biomarkers through their antioxidant, anti-inflammatory and metabolic effects [20,23,24].

A promising nutritional intervention that has been gaining traction recently is the use of postbiotics, which in 2021 were defined by ISAPP as “*a preparation of inanimate microorganisms and/or their components that confers a health benefit on the host*” [24]. Postbiotics have recently gained interest not only because they have functional activity [25] but also because their inactivation allows for incorporation into a wider range of food applications in which their use was previously limited, such as isotonic drinks and sports bars, energy gels or carbonated beverages [26,27]. However, evidence on the role of postbiotics in mitigating exercise-induced side effects remains limited [28]. Indeed, to our knowledge, among the few clinical studies currently available, only one clinical trial has reported an improvement in an oxidative stress biomarker following dietary supplementation with a postbiotic (heat-treated *L. paracasei* PS23) [29].

In a randomized placebo-controlled clinical trial, the effect of a combination of three probiotic strains, *B. longum* CECT 7347 (Esflorin1™), *L. rhamnosus* CECT 8361 (BPL15) and *L. casei* CECT 9104 (BPL4), all isolated from a healthy breast-fed infant, was assessed in a model of exercise-induced oxidative stress in male cyclists [30]. The probiotic blend, with the commercial name Active Lifestyle, reduced several oxidative stress biomarkers in healthy subjects after acute high-intensity exercise [30]. Even though the probiotic blend demonstrated a clear antioxidant effect, it remains unclear whether this activity would persist after heat treatment. Previous studies with Esflorin1™ probiotic (*B. longum* CECT 7347) have shown that its heat-treated postbiotic form retained anti-inflammatory and antioxidant activities in cell culture in vitro and in the *C. elegans* model and improved intestinal permeability [31]. Similarly, it has been observed that heat-treated versions of other *Bifidobacterium* and *Lactobacillus* strains maintain their immunomodulatory effects [32,33] on exercise performance [34] and even lipid-lowering activities [35] in preclinical studies. However, the impact of heat treatment on probiotic functionality is treatment- and strain-dependent, as, for instance, heat-inactivated LGG and BB12 exhibit reduced barrier-enhancing effects compared with their viable counterparts [36].

The aim of this study was to investigate the functional effects of the probiotic blend in the context of exercise-associated side effects and to determine whether these effects are preserved after heat treatment. Accordingly, in vitro models (radical scavenging and cell cultures) and a *C. elegans* model were used to evaluate biomarkers associated with antioxidant capacity, immunomodulation and gut-barrier protection. Furthermore, we sought to provide mechanistic insights into the actions of both blends, focusing on pathways relevant to oxidative stress management and energy production.

## 2. Materials and Methods

### 2.1. Bacterial Strains

Active Lifestyle from ADM Biopolis, S.L. (Paterna, Spain) was used in this study in both probiotic and heat-treated forms. This blend is composed of *B. longum* CECT 7347 (Esflorin1™), *L. rhamnosus* CECT 8361 (BPL15) and *L. casei* CECT 9104 (BPL4) strains. Strains were cultured overnight at 37 °C in Man, Rogosa and Sharpe (MRS) broth (Scharlab, Sentmenat, Spain) supplemented with 0.05% (*w*/*v*) cysteine hydrochloride (Coralim aditivos SL, Paterna, Spain) under anaerobic conditions. Heat-treated (HT) preparations were obtained by harvesting bacterial cells by centrifugation, washing them with sterile 0.9% (*w*/*v*) NaCl solution and subsequently autoclaving the suspension at 121 °C for 20 min. Live and HT bacterial preparations were quantified using a CytoFLEX flow cytometer (Beckman Coulter, Suzhou, China).

### 2.2. Cell Cultures

The human colonic epithelial cell line HT-29 (ATCC HTB-38™, American Type Culture Collection; Rockville, MD, USA) was cultured as monolayers at 37 °C and 5% CO_2_ in McCoy’s 5A medium with 10% fetal bovine serum (FBS) containing streptomycin (100 µg/mL) and penicillin (100 IU/mL).

Caco-2 human colonic epithelial cells (ECACC 86010202, European Collection of Authenticated Cell Cultures (Salisbury, UK)) were grown in high-glucose (25 mM) Dulbecco’s Modified Eagle’s Medium (DMEM) supplemented with 10% FBS, 2 mM L-Glutamine, 1% non-essential amino acids, 100 μg/mL streptomycin and 100 IU/mL penicillin.

The human monocyte cell line U-937 (ATCC CRL-1593.2™) was cultured in 96-well plates (1 × 10^5^ cells/well) in RPMI 1640 medium with 10% FBS, 2 mM L-Glutamine and streptomycin (100 µg/mL) and penicillin (100 IU/mL) and differentiated into macrophages through incubation with 50 ng/mL phorbol 12-myristate 13-acetate (PMA) for 72 h at 37 °C and 5% CO_2_.

Murine C2C12 myoblasts (ATCC CRL-1458™) were routinely maintained in high-glucose DMEM medium supplemented with 10% FBS, 2 mM L-Glutamine, streptomycin (100 µg/mL) and penicillin (100 IU/mL).

All cell culture reagents were obtained from Thermo Fisher (Waltham, MA, USA).

### 2.3. C. elegans Strains and Maintenance Conditions

*C. elegans* strains N2 Bristol (wild-type) and CF1553 (muIs84 [(pAD76) sod-3p::GFP + rol-6(su1006)]) were obtained from the Caenorhabditis Genetics Center (CGC) at the University of Minnesota (Minneapolis, MN, USA) and maintained at 20 °C on nematode growth medium (NGM) plates with *Escherichia coli* strain OP50 as a normal diet for nematodes [37].

### 2.4. Radical Scavenging Activity

The radical scavenging activity of the probiotic or heat-treated blend was determined using the 2,2-diphenyl-1-picrylhydrazyl (DPPH) method. Cell concentrations were first standardized in sterile 1X PBS to 1 × 10^8^ CFU/mL. For the heat-treated blend, heat treatment was performed after this standardization step. Subsequently, probiotic and heat-treated blend preparations were centrifuged, the PBS was discarded, and the resulting pellets were resuspended in 500 μL of methanol. Then, 500 μL of 0.2 mM DPPH solution (2D9132; Merck; Darmstadt, Germany) was added to each sample. DPPH with or without ascorbic acid (10 µg/mL) were used as positive and negative controls, respectively. Samples were incubated in the dark at room temperature for 30 min. DPPH scavenging was monitored by measuring the absorbance at 517 nm in a Multiskan SkyHigh spectrophotometer (Thermo Fisher; Waltham, MA, USA). Radical scavenging activity was quantified as follows: (Abs C − Abs S)/Abs C = % of inhibition, where “Abs C” is the absorbance value of negative control and “Abs S” is the absorbance value of the sample. Data are presented as the average of three independent experiments in duplicate.

### 2.5. Oxidative Stress Resistance in C. elegans

*C. elegans* wild-type strain N2 was egg-synchronized in NGM plates with *E. coli* OP50 (control medium) alone or containing the probiotic or heat-treated blend at a total dose of 1 × 10^8^ CFU/plate or 1 × 10^8^ cells/plate, respectively. Nematode viability was assessed after 4 h of oxidative stress induction with 2 mM H_2_O_2_ (Merck), as previously described [38]. Vitamin C (10 µg/mL, Merck) was used as positive control. Experiments were carried out in triplicate (150 worms/condition).

### 2.6. Intracellular Reactive Oxygen Species in H_2_O_2_ Exposed C. elegans

Intracellular ROS in *C. elegans* was measured using the fluorescent probe 2′,7′-dichlorofluorescein diacetate (H2DCFDA; Merck). Synchronized L4 nematodes were cultured on NGM plates with *E. coli* OP50 (control medium) alone or containing the probiotic (1x10^8^ CFU/plate) or heat-treated blend (1 × 10^8^ cells/plate) at 20 °C. Vitamin C (10 µg/mL) was used as positive control. Once worms reached the 5 days of adult age, they were exposed to 2 mM H_2_O_2_ for 4 h at 20 °C. Then, worms were collected, washed and treated with a 25 μM H2DCFDA solution. Samples were read in a fluorescent microplate reader (Synergy H1, Biotek; Winooski, VT, USA) using excitation at 485 nm and emission at 535 nm. Four independent experiments were performed (500–600 worms/condition).

### 2.7. Antioxidant Sod-3 Reporter Line Expression in C. elegans

Sod-3 expression was measured by quantifying the fluorescence of the green fluorescent protein (gfp) reporter. Synchronized nematodes of the *C. elegans* reporter strain CF1553 were cultured on NGM plates with *E. coli* OP50 (control medium) alone or containing the probiotic (1 × 10^8^ CFU/plate) or heat-treated blend (1 × 10^8^ cells/plate) at 20 °C. Once worms reached 5 days of adult age, the fluorescence intensity was analyzed with the Nikon SMZ18 stereomicroscope equipped with NIS-ELEMENT v6. image software (Nikon Instruments Inc.; Melville, NY, USA). Four independent assays were performed (replicates) with 20 worms per condition. Folic acid (50 µM) was included as positive control. The results are shown as the percentage of fluorescence of each worm (a total of 80 worms/condition) in each condition with respect to NGM.

### 2.8. Mitochondrial Staining and Quantification in C2C12 Myotubes

C2C12 myoblasts were seeded at 1 × 10^5^ cells/well in 6-well plates and cultured until reaching confluence (48 h). To differentiate myoblasts into myotubes, the cell culture medium was replaced by high-glucose DMEM supplemented with 2% heat-inactivated horse serum (Thermo Fisher), 2 mM L-Glutamine, streptomycin (100 µg/mL) and penicillin (100 IU/mL) and cultured for five days, when myotubes were fully formed.

Mitochondrial content was determined in the C2C12 myotubes, using the 10-N-nonyl acridine orange (NAO) staining method (Sigma, St. Louis, MO, USA). Probiotic or heat-treated blend was added to the C2C12 myotubes at concentrations of 1 × 10^8^ CFU/mL and 1 × 10^9^ cells/mL, respectively, and incubated for 24 h at 37 °C in a 5% CO_2_ atmosphere using antibiotic-free media. Resveratrol (100 µM, Merck) was included as positive control for mitochondrial biogenesis activation. After the treatments, cells were harvested by trypsinization and stained with NAO (100 nM, Merck) for 30 min. Excess dye was removed by washing the cells with Dulbecco’s phosphate-buffered saline (dPBS, D8537, Sigma-Aldrich), and cell fluorescence was recorded using a CytoFLEX flow cytometer (PC5.5 channel). For each condition, 1 × 10^4^ cells were analyzed to determine the median fluorescence intensity (MFI).

### 2.9. Intracellular ATP Content in C2C12 Myotubes

To determine intracellular energy in C2C12 myotubes, probiotic (1 × 10^8^ CFU/mL) or the heat-treated blend (1 × 10^9^ cells/mL) was added to the C2C12 myotubes and incubated for 24 h at 37 °C in a 5% CO_2_ atmosphere in antibiotic-free media. Resveratrol (100 µM) was included as positive control. Cells were washed twice with dPBS and harvested by trypsinization. Cells were then lysed using Pierce™ Luciferase Cell Lysis Buffer containing protease and phosphatase inhibitor cocktail (Thermo Fisher). Cell extracts were protein-quantified by the bicinchoninic acid protein assay (BCA, Thermo Fisher). Adenosine 5′-triphosphate (ATP) was measured with the ATP Determination Kit (Thermo Fisher, A22066) according to the manufacturer’s instructions using 10 µg of cell lysates. Luminescence was recorded in a Synergy H1 multiplate reader, and ATP was determined by interpolation from a calibration curve using ATP as a standard.

### 2.10. AMPK Signaling Pathway in C2C12 Myotubes

On the day of the experiment, C2C12 myotubes were subjected to starvation for 3 h in low-glucose (5 mM) DMEM supplemented with 2% heat-inactivated horse serum, 2 mM L-Glutamine and streptomycin (100 µg/mL) and penicillin (100 IU/mL). Probiotic or heat-treated blend was added to C2C12 myotubes at 1 × 10^8^ CFU/mL and 1 × 10^9^ cells/mL, respectively, and incubated for 5 h at 37 °C and 5% CO_2_ in antibiotic-free media. 5-Aminoimidazole-4-carboxamide ribonucleotide (AICAR, 2 mM, Merck) was used as a control for AMPK activation. After treatment, cells were washed twice in cold PBS and harvested by scraping. Cells were then lysed in cell extraction buffer supplemented with protease and phosphatase inhibitor cocktail by incubation for 30 min with vortexing at 10 min intervals on ice. Then, samples were centrifuged at 13,000 rpm for 10 min at 4 °C and supernatants were quantified for protein content by the BCA method as described before and stored at −80 °C until analysis. AMPKα protein levels phosphorylated at threonine residue 172 were measured as a readout of AMPK activation by an ELISA kit (AMPKα p T172, Thermo Fisher) according to the manufacturer’s instructions using 40 µg of total protein/sample. Results are displayed as the percentage of AMPKα [pT172] content in each treatment with respect to control untreated cells.

### 2.11. Anti-Inflammatory Activity in HT-29 Cells

To evaluate IL-8 secretion, HT-29 cells were seeded at 5 × 10^4^ cells/well in 96-well plates and cultured for 7 days to differentiate. Then, TNF-α (Sigma-Aldrich; St. Louis, MO, USA) (4 ng/mL) was added to induce IL-8 release in the presence or absence of the probiotic (1 × 10^8^ CFU/mL) or heat-treated (1 × 10^9^ cells/mL) blend in antibiotic-free medium for 3 h. Complete McCoy’s 5A medium was used as a negative control. IL-8 concentrations in culture media were quantified using an IL-8 (CXCL8) Human ProcartaPlex™ Simplex Kit (Thermo Fisher), according to the manufacturer’s instructions and with the Luminex 200™ System (DiaSorin; Saluggia, Italy). Cell viability was determined by 3-(4,5-dimethylthiazol-2-yl)-2,5-diphenyltetrazolium bromide (MTT) assay (Abcam; Cambridge, UK).

### 2.12. Transepithelial Electrical Resistance (TEER) Assay in Caco-2 Cells

Caco-2 cells were seeded in 24-well insert plates (1 μm pore size, polyethylene terephthalate, 0.7 cm^2^ surface area, Merck) at 5 × 10^4^ cells/insert. Cell culture media was refreshed every 3–4 days and inserts were maintained with 400 μL in the apical compartment and 800 μL in the basal compartment. Tight junction formation in the cell cultures was monitored daily by the measurement of transepithelial electrical resistance (TEER) with an ERS-2 Voltohmmeter EVOM (Merck).

Permeability assays were performed on day 10 post-seeding, when Caco-2 monolayers exhibited TEER values of approximately 1000–1500 Ω cm^2^, indicative of fully developed tight junctions, as previously reported [39]. On the day of the experiment, the probiotic or heat-treated blend was resuspended in complete medium without antibiotics at a final concentration of 1 × 10^8^ CFU/mL and 1 × 10^9^ cells/mL, respectively, and added to the apical side of the insert. After incubating at 37 °C for 1 h, TEER was measured to confirm monolayer integrity (initial value). Subsequently, H_2_O_2_ (0.3 mM) was added to the apical compartment, and the plate was incubated for an additional hour at 37 °C. Finally, TEER was re-measured, and relative TEER variation was calculated. Results are shown as the average of three independent experiments (replicates), each performed with technical triplicates.

### 2.13. Intestinal Barrier Integrity in C. elegans

Wild-type strain (N2) synchronized worms were cultured on NGM plates seeded with OP50 (control) or supplemented either with probiotic or heat-treated blend at a final concentration of 1 × 10^8^ CFU/plate or 1 × 10^8^ cells/plate, respectively. L4 nematodes were exposed to 0.5 µg/mL methotrexate (MTX; Sigma-Aldrich; St. Louis, MO, USA) for 24 h to induce intestinal permeability. Nile Red staining (0.05 µg/mL) was used to evaluate intestinal permeability. A condition without intestinal damage (NGM control) was included. Three independent assays were performed, each with 30 worms per condition, randomly selected and analyzed using a Nikon SMZ18 fluorescence stereomicroscope equipped with NIS-ELEMENT image software (Nikon Instruments Inc.). Results are expressed as fluorescence percentage per worm (90 worms per condition in total) relative to the untreated nematode control (without MTX-induced damage).

### 2.14. Immunomodulation in U-937 Macrophages

To study immunomodulation properties, macrophages were stimulated with probiotic (1 × 10^8^ CFU/mL) or heat-treated blend (1 × 10^9^ cells/mL), respectively, or 5 µg/mL LPS (*Escherichia coli* O55:B5, Merck) in complete RPMI 1640 medium without antibiotics for 5 h at 37 °C and 5% CO_2_. Cell supernatants were collected and stored at −20 °C until further analysis. TNF-α, IL-1β, IL-18, IL-8, IL-6, IL-10, Interleukin 1 Receptor Antagonist (IL-1RA), Granulocyte Colony-Stimulating Factor (G-CSF), granulocyte-macrophage colony stimulating factor (GM-CSF), and epidermal growth factor (EGF) concentrations were measured using the Luminex 200™ System. The ProcartaPlex™ Mix and Match commercial kit (Thermo Fisher) was used according to the manufacturer’s instructions.

### 2.15. Statistical Analysis

Results are expressed as the mean ± standard deviation (SD). Data normality was assessed using the Shapiro–Wilk/Kolmogorov–Smirnov tests. Depending on whether the data followed a normal distribution, the appropriate parametric or non-parametric statistical tests were applied. In addition, prior to the parametric tests, a Brown–Forsythe test was applied to verify that there were no differences in variance.

A one-way analysis of variance (ANOVA) followed by Tukey’s multiple comparisons test was used for data analysis. This approach was applied for in vitro radical scavenging activity, ROS levels and survival in *C. elegans*, anti-inflammatory activity in HT-29 cells and gut permeability in Caco-2 cells. RM one-way ANOVA with Tukey’s multiple comparison post-test was used for mitochondrial content, ATP levels and AMPK activation in C2C12. For sod-3 expression and gut permeability in *C. elegans*, the Kruskal–Wallis followed by Dunn’s multiple comparisons test was applied. For the immunomodulation activity in U-937 macrophages, data was analyzed using either one-way ANOVA followed by Tukey’s multiple comparisons test or Kruskal–Wallis with Dunn’s multiple comparisons test, depending on data distribution. All the analyses were performed with GraphPad Prism 10 software (GraphPad Software; Boston, MA, USA). A *p*-value lower than 0.05 was considered statistically significant.

## 3. Results

### 3.1. Both Heat-Treated and Probiotic Blends Have Antioxidant Effects

The probiotic blend exerted antioxidant effects in male cyclists exposed to high-intensity physical exercise [30]. Therefore, intracellular ROS levels and the survival of *C. elegans* (wild-type N2) exposed to H_2_O_2_-induced oxidative stress were evaluated following supplementation with either blend.

**Figure 1:**
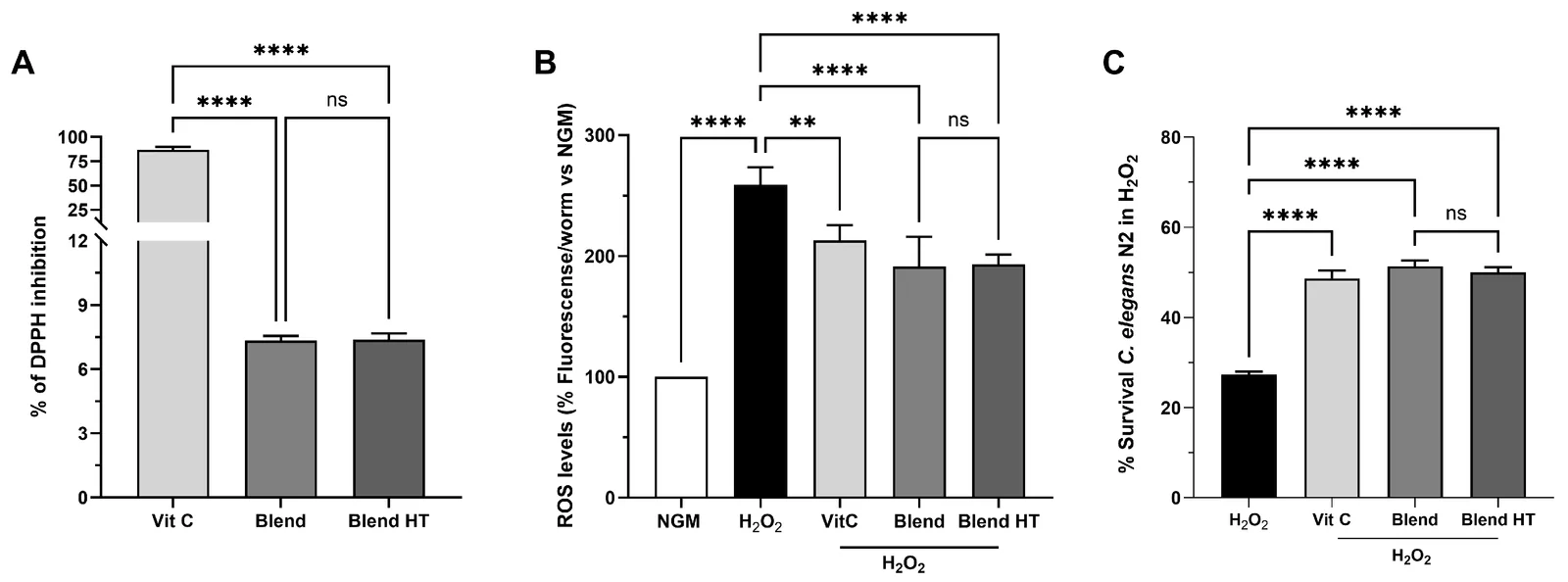
Antioxidant effects of the probiotic and heat-treated blends. Radical scavenging activity expressed as % DPPH inhibition of the probiotic and heat-treated blends and vitamin C (10 µg/mL) ((**A**), n = 3 independent experiments), intracellular ROS levels expressed as % fluorescence per nematode ((**B**), n = 3 independent experiments; 150 worms/condition) and survival of *C. elegans* fed with vitamin C (10 µg/mL) and the probiotic or heat-treated blends, after acute oxidative stress induced with H_2_O_2_ ((**C**), n = 4 independent experiments; 500–600 worms/condition). Data are represented as the mean ± SD. Statistical tests: one-way ANOVA with Tukey’s multiple comparisons post-test. ** *p* ≤ 0.01; **** *p* ≤ 0.0001. HT, heat-treated; NGM, nematode growth medium; ns, not significant; Vit C, vitamin C.

To evaluate the antioxidant potential of the heat-treated blend, its radical scavenging activity was determined using the DPPH assay and compared with that of the probiotic blend. The probiotic and heat-treated blends provided 7.5 ± 0.15% and 7.5 ± 0.20% DPPH radical scavenging activity inhibition, respectively, with no statistically significant difference between both forms (Figure 1A). The positive control vitamin C inhibited 86.4 ± 3.4% the DPPH radical scavenging activity. This antioxidant effect was statistically significant compared to both the probiotic and heat-treated blends (*p* < 0.0001 for both).

In the *C. elegans* model, the probiotic and heat-treated blends reduced intracellular ROS levels comparably (67.5 ± 24.6% and 65.8 ± 8.1%, respectively, compared with the H_2_O_2_ condition). In both cases, there was a statistically significant reduction in ROS levels, in comparison with the H_2_O_2_ control (*p* < 0.0001 for both) (Figure 1B). These reductions in intracellular ROS were associated with increased survival in nematodes fed with probiotic or heat-treated blends under oxidative stress, compared with control-fed nematodes (increase of 24.0 ± 2.0% and 23.3 ± 1.2%, respectively; *p* < 0.0001 for both), and with no statistical differences between the two blends (Figure 1C).

**Figure 2:**
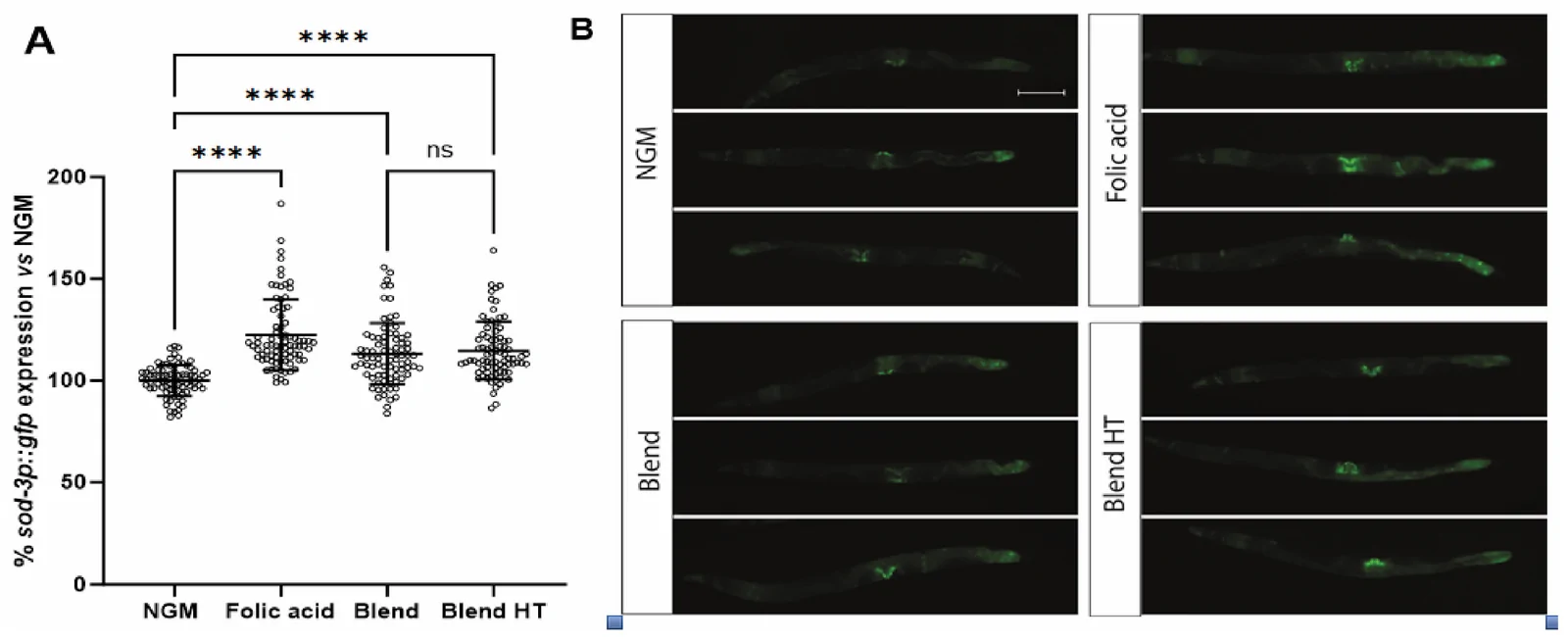
Effects on antioxidant enzyme gene expression of the probiotic and heat-treated blends. Sod-3p::gfp expression quantification expressed as % relative to NGM control condition ((**A**), n = 4 independent experiments; 80 worms/condition), and representative fluorescence images of *C. elegans* fed with folic acid (50µM) and the probiotic or heat-treated blends (**B**). Data are represented as the mean ± SD. Statistical tests: Kruskall–Wallis with Dunn’s multiple comparisons test was applied considering all individual data points. **** *p* = 0.0001. HT, heat-treated; NGM, nematode growth medium; ns, not significant. Scale bar = 500 µm.

After confirming that feeding *C. elegans* with the heat-treated blend reduced intracellular ROS levels and improved survival under H_2_O_2_-induced oxidative stress, we investigated the molecular mechanism involved in the stress modulation. With this goal, antioxidant enzyme *sod-3* expression was measured using the gfp transgenic reporter *C. elegans* CF1553 strain [40]. Both the probiotic and heat-treated blends significantly induced *sod-3* gfp expression (13.1 ± 2.1% and 14.7 ± 1.5%, respectively), compared to the control condition (NGM) (*p* < 0.0001 for both) (Figure 2). The positive control, folic acid, increased sod-3 gfp expression in CF1553 *C. elegans* by 22.5 ± 5% (*p* < 0.0001 vs. NGM). No significant differences were observed when comparing folic acid with either the probiotic or heat-treated blends (Figure 2).

Therefore, both the heat-treated and the probiotic blends exhibited antioxidant activity, with no significant differences between them, resulting in increased nematode survival under oxidative stress. Furthermore, their capacity to induce *sod-3* expression corroborated the antioxidant effect of the blends under non-stress conditions.

### 3.2. Mitochondrial and ATP Content Increased by Heat-Treated Blend in C2C12 Myotubes

**Figure 3:**
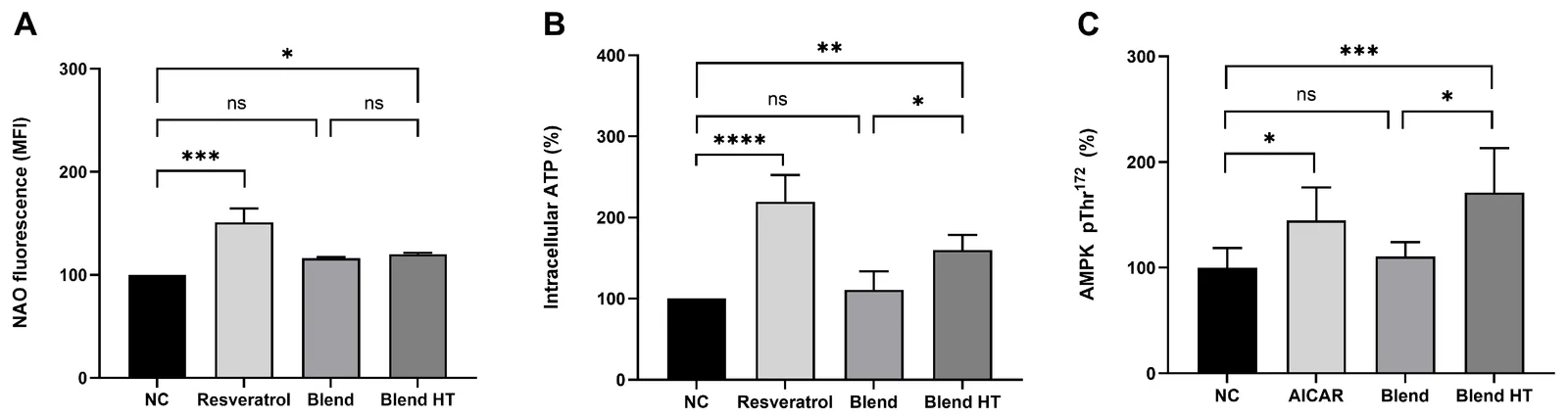
Mitochondrial content, intracellular ATP levels and AMPK activation in muscular cells upon treatment with probiotic and heat-treated blend. NAO (10-N-nonyl acridine orange) fluorescence ((**A**), n = 3 independent experiments), intracellular ATP content ((**B**), n = 4 independent experiments) and AMPK phosphorylation (pThr^172^) ((**C**), n = 5 independent experiments) in C2C12 myotubes incubated with the probiotic or the heat-treated blends. Data are represented as the mean ± SD. Statistical tests: RM one-way ANOVA with Tukey’s multiple comparison post-test for mitochondrial content and intracellular ATP assay, and Brown–Forsythe and Welch ANOVA assay with Dunnett’s multiple comparison post-test for AMPK phosphorylation. * *p* ≤ 0.05; ** *p* ≤ 0.01; *** *p* ≤ 0.001; **** *p* ≤ 0.001. AICAR, 5-Aminoimidazole-4-carboxamide ribonucleoside; AMPK, AMP-activated protein kinase; HT, heat-treated; NC, negative control; ns, not significant.

To evaluate whether the heat-treated and probiotic blend influence mitochondrial biogenesis, mitochondrial biomass was quantified in C2C12 myotubes using NAO dye. The heat-treated blend significantly increased the mitochondrial content by 20.0 ± 1.4% compared to the negative control (*p* < 0.05). The probiotic blend showed a similar trend, increasing mitochondrial content by 16.3 ± 1.0%, although this did not reach statistical significance (*p* = 0.09) (Figure 3A). As expected, the positive control, resveratrol, increased mitochondrial biogenesis (50.5 ± 13.6%, *p* < 0.001 vs. negative control) (Figure 3A).

To assess whether the blends might influence energy status, the effects of the heat-treated blend, the probiotic blend and resveratrol (as a positive control) on total ATP content were measured in the C2C12 myotubes. As shown in Figure 3B, the positive control, resveratrol, increased ATP content by 119.6 ± 32.7% compared to the negative control (*p* < 0.0001). The heat-treated blend also increased ATP content by 59.9 ± 18.5% compared to the negative control (*p* < 0.01). The probiotic blend did not significantly increase ATP content (11.0 ± 22.7%) compared to negative control (*p* > 0.05), at the evaluated concentration.

### 3.3. The Heat-Treated Blend Increased AMPK Phosphorylation in C2C12 Myotubes

To investigate the underlying mechanism of action of the heat-treated and probiotic blends, AMPK phosphorylation was evaluated in C2C12 myotubes as an indicator of mitochondrial and ATP content, and compared with AICAR, an AMPK agonist that mimics exercise.

As shown in Figure 3C, the heat-treated blend increased AMPK phosphorylation in C2C12 myotubes by 71.3 ± 42.0% compared with the negative control (*p* < 0.001). AICAR, used as a positive control, also increased AMPK phosphorylation by 44.7 ± 31.3% (*p* < 0.05). No significant differences were observed between the heat-treated blend and AICAR (*p* = 0.54), suggesting comparable statistical effects rather than equivalent effect magnitudes. In contrast, the probiotic blend did not significantly increase AMPK phosphorylation compared with the negative control (10.6 ± 13.7%; *p* > 0.05) at the evaluated dose.

Overall, the heat-treated blend markedly enhanced mitochondrial content, ATP levels and AMPK phosphorylation in C2C12 myotubes, whereas the probiotic blend did not induce statistically significant changes at the concentrations evaluated.

### 3.4. Anti-Inflammatory Effect in HT-29 Cells of Heat-Treated and Probiotic Blends

Exhaustive or unaccustomed exercise may lead to a temporary increase in intestinal permeability and inflammation, including secretion of IL-8, due to reduced blood flow [41]. Therefore, the effect of the heat-treated and probiotic blends on IL-8 secretion were evaluated in the HT-29 intestinal cell line exposed to a pro-inflammatory stimulus.

**Figure 4:**
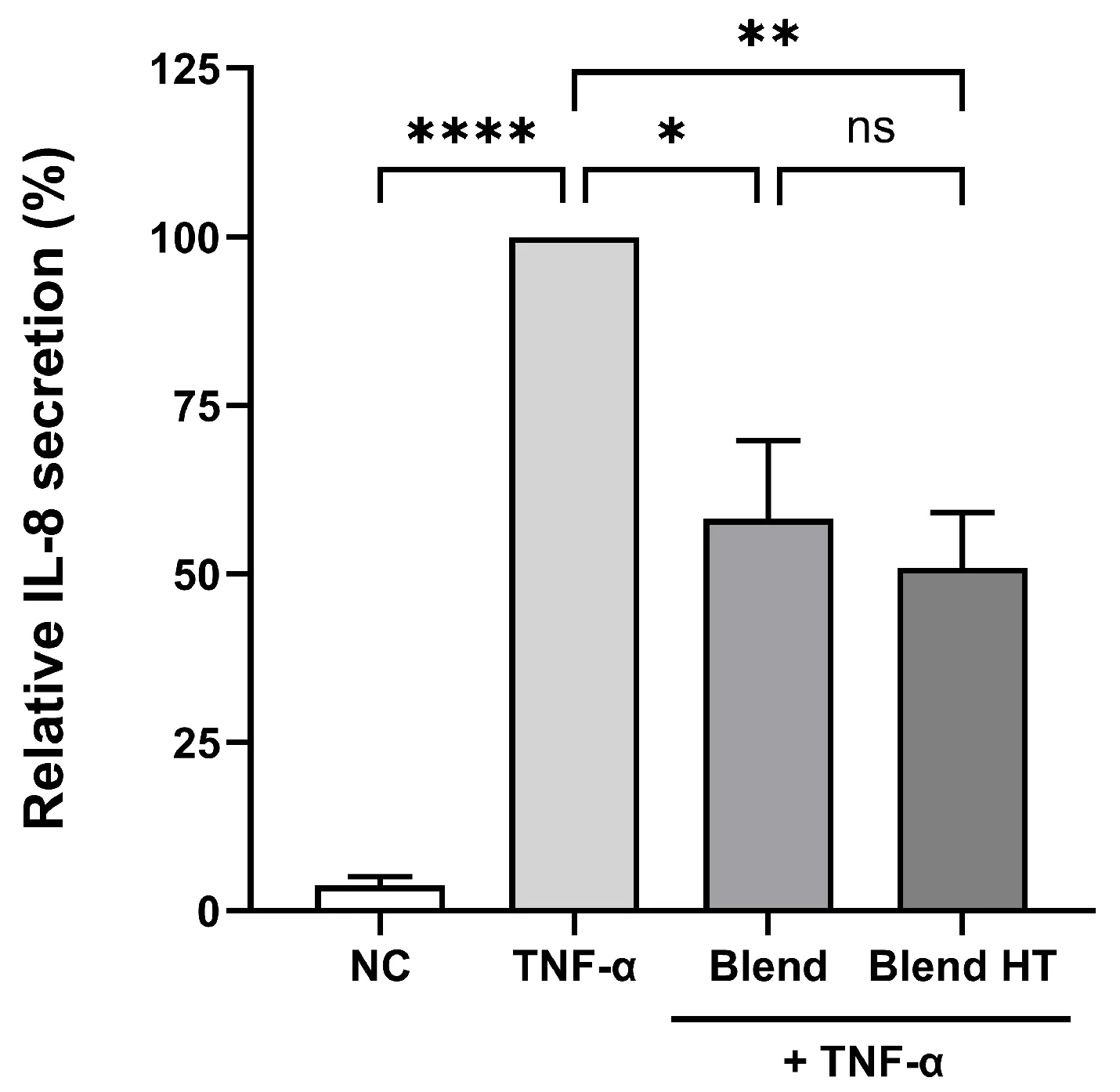
Anti-inflammatory activity of the probiotic and heat-treated blends. Interleukin 8 released by HT-29 cells incubated with the probiotics or heat-treated blends in presence of TNF-α (n = 4 independent experiments). Data are represented as the mean ± SD. Statistical tests: RM one-way ANOVA with Tukey’s multiple comparisons test: * *p* ≤ 0.05; ** *p* ≤ 0.01 and **** *p* ≤ 0.0001. HT, heat-treated; ns, not significant; TNF-α, tumoral necrosis factor α.

As shown in Figure 4, the pro-inflammatory stimulus with TNF-α increased IL-8 release in HT-29 cells (*p* < 0.0001 vs. negative control). Both the heat-treated and probiotic blends significantly reduced IL-8 secretion in TNF-α-stimulated cells (41.8 ± 11.5% (*p* < 0.01) and 49.1 ± 8.2% (*p* < 0.05)), respectively, vs. TNF-α-stimulated cells alone). No significant differences were observed between the blends, suggesting a similar anti-inflammatory effect at the tested doses.

### 3.5. Intestinal Barrier Integrity Preservation by the Heat-Treated and Probiotic Blends in Caco-2 Cells and Caenorhabditis elegans

After confirming the reduction of IL-8 secretion in HT-29 cells, we evaluated whether the blends could protect the intestinal epithelium. To this end, we assessed if administration of the blends to Caco-2 cells and the nematode *C. elegans* reduced intestinal permeability induced by either H_2_O_2_ or methotrexate, respectively.

**Figure 5:**
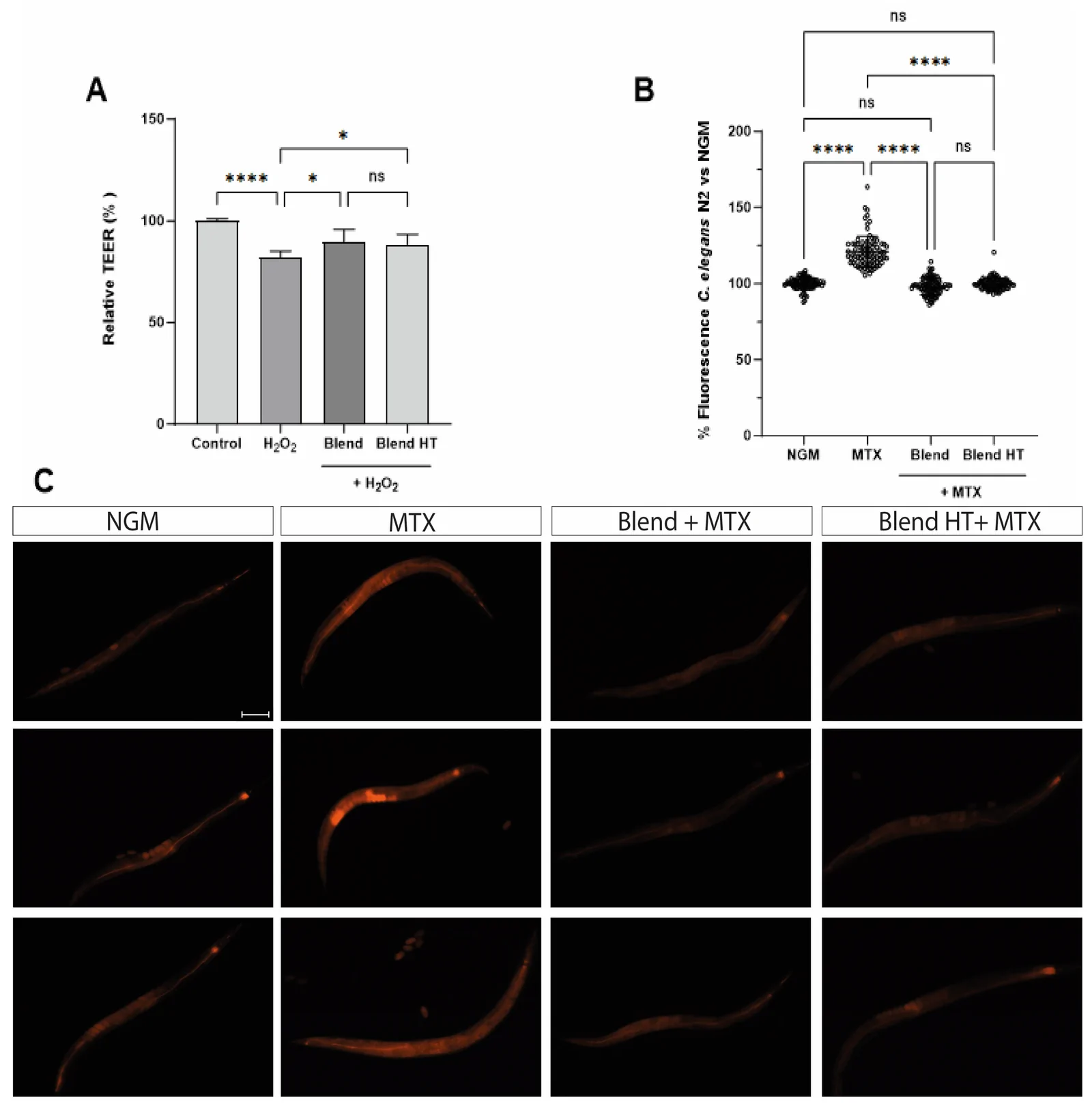
Gut permeability protection effect of the probiotic and heat-treated blends. Relative transepithelial electrical resistance (TEER) damage after 1 h of H_2_O_2_ exposure in the presence or absence of probiotic and heat-treated blends in Caco-2 cells ((**A**), n = 8 independent experiments). Percentage of fluorescence intensity of Nile Red vs. NGM in worms treated with MTX (0.5 µg/mL) and the probiotic and heat-treated blends. ((**B**), n = 3 independent experiments). Representative images of Nile Red staining in live young adult *C. elegans* N2 worms under fluorescence microscopy. Worms were treated with the probiotic and heat-treated blends, and intestinal damage was induced with MTX (**C**). Statistical tests: one-way ANOVA with Tukey’s multiple comparisons test for TEER and the Kruskal–Wallis test with Dunn’s multiple comparisons test for fluorescence intensity. * *p* ≤ 0.05; **** *p* ≤ 0.0001. HT, heat-treated; MTX, methotrexate; NGM, nematode growth media; ns, not significant. Scale bar = 100 μm.

Results showed that H_2_O_2_ promoted epithelial damage, as shown by a decrease in the TEER values of 18.9 ± 3.1% compared to untreated Caco-2 cells (*p* < 0.0001). Regarding the heat-treated and probiotic blends, both exerted protective effects on permeability, reducing the TEER damage by 30.1 ± 10.5% and 33.3 ± 11.1%, respectively, compared to the Caco-2 cells treated with H_2_O_2_ (*p* < 0.05 in both cases) (Figure 5A).

MTX increased intestinal permeability of *C. elegans* compared to the control conditions, with an increase of 20.9 ± 3.2% in Nile Red fluorescence (*p* < 0.0001). Both heat-treated and probiotic blends prevented the MTX-induced intestinal permeability increase, with a reduction in fluorescence produced by Nile Red staining of 21.0 ± 2.4% and 22.9 ± 1.1%, respectively, compared to MTX (*p* < 0.0001 for both) (Figure 5B). Representative images of nematodes in each condition, obtained by fluorescence microscopy, are shown in Figure 5C.

In summary, there were no differences between the protection exerted by the probiotic and heat-treated blends in either intestinal permeability model.

### 3.6. Heat-Treated and Probiotic Blends Immunomodulatory Effect in U937 Macrophages

Several studies have shown that prolonged, high-intensity physical activity transiently suppresses immune function in athletes, thereby increasing susceptibility to upper respiratory tract infections [8,19,42]. Therefore, we evaluated the immunomodulatory effects of the probiotic and heat-treated blends on U937 macrophages. All results regarding the immunomodulatory effects on U937 macrophages are shown in Table 1.

Both the probiotic and heat-treated blends significantly increased the release of all measured cytokines compared to the negative control, including not only the pro-inflammatory cytokines (TNF-α, IL-1β and IL-18) but also the regulatory (IL-6, G-CSF, GM-CSF) and the anti-inflammatory cytokines (IL-10, IL-RA, EGF). This indicates a broad immunomodulatory effect on the macrophages for both blends.

## 4. Discussion

Although moderate physical activity confers numerous health benefits, high-intensity exercise can promote the generation of ROS, leading to oxidative damage of cellular components such as nucleic acids, proteins and lipids. The resulting oxidative stress is also considered a potential cause of myocyte membrane damage, which may trigger an exacerbated inflammatory response. These responses not only have a negative impact on skeletal muscle but might also increase digestive discomfort and potentially weaken the immune system.

In this sense, numerous nutritional interventions have tested the effect of probiotics in alleviating oxidative stress and other adverse effects induced by unaccustomed or high-intensity exercise [43]. In a previous study, the effect of a 6-week intake of the studied probiotic blend (*B. longum* CECT 7347, *L. rhamnosus* CECT 8361 and *L. casei* CECT 9104) was evaluated on an oxidative stress model of high-intensity and duration exercise in male cyclists [30]. In that clinical study, biomarkers related to lipid- and DNA-related oxidative stress such as MDA, Ox-LDL and 8-OHdg were improved. However, limited information is available on the functional activity of the heat-treated form of this blend and the specific cellular and molecular mechanisms underlying its antioxidant effect and recovery potential in the context of exercise.

The results of this study corroborate, in a pre-clinical setting, the antioxidant activity previously observed in the clinical trial. Although both the probiotic and heat-treated blends exhibited statistically significant DPPH radical scavenging activity, their effects were modest compared with that of vitamin C. Therefore, the effects observed in subsequent assays are likely attributable primarily to the modulation of cellular redox homeostasis and antioxidant defense mechanisms. Nevertheless, the Active Lifestyle heat-treated blend preserves the antioxidant effect of the probiotic formulation, as evidenced by the decrease in intracellular ROS and enhanced survival of *C. elegans* under acute oxidative stress, with effects comparable to those of the probiotic blend. This antioxidant response might be mediated through the upregulation of *sod-3* expression, as observed in the CF1553 C. elegans strain [44,45]. This gene encodes a mitochondrial superoxide dismutase (SOD-3) that helps convert superoxide radicals (O_2_^−^) into hydrogen peroxide and oxygen, thereby contributing to cellular antioxidant defense. Furthermore, SOD-3 enzyme is part of the muscle adaptative response to exercise, and its levels increase in response to exercise both in *C. elegans* and in humans [44,45,46]. In agreement with our results, previous studies have reported that specific probiotics increase the gene expression of *sod-3* in nematodes. Similarly, other studies have shown that probiotics [47,48,49] and their derived compounds (secreted metabolites [50] and structural molecules) [51,52,53,54,55] have antioxidant effects in both in vitro and in vivo (*C. elegans*) models. However, there are fewer studies that have evaluated the antioxidant activity of heat-treated strains [31,56,57,58,59]. Given that *C. elegans* is a well-established model for studding oxidative stress and redox signaling pathways relevant to human physiology [59], these findings provide mechanistic support for the functionality of the heat-treated blend; however, clinical studies are needed to determine whether these effects translate to humans.

The heat-treated blend significantly increased AMPK phosphorylation in C2C12 myotubes, a key regulator of substrate uptake, oxidative metabolism and mitochondrial biogenesis [60]. In contrast, the probiotic blend did not induce detectable changes under the conditions tested on AMPK phosphorylation. However, these findings do not exclude the possibility that the probiotic blend may exert more pronounced biological effects under different experimental settings, including alternative doses, exposure times or cellular models. The assessment of higher concentrations was limited by the potential negative effects of excessive bacterial loads on host cell viability.

These findings are consistent with previous reports describing AMPK activation following exposure to microbial-derived bioactive compounds. In particular, Toda K et al. observed higher AMPK phosphorylation in the soleus of rats fed with the heat-treated *B. breve* B-3 compared to its viable form [61]. Likewise, among studies performed in C2C12 myotubes [62,63,64], Cai et al. observed an increase in AMPK phosphorylation, mitochondrial biogenesis and enhanced ATP content in C2C12 myotubes incubated with *Yamadazyma* triangularis XHY69-derived peptides [65]. However, evidence linking heat-treated bacterial preparations to these metabolic responses remains scarce.

While AMPK activation induced by probiotics has frequently been associated with microbial production of short-chain fatty acids (SCFAs) [66], the response observed in vitro with the heat-treated blend suggests that alternative mechanisms may be involved. Changes in cellular energy status, reflected by increased AMP/ATP and ADP/ATP ratios, are among the primary activators of AMPK [67]. In this context, a recent study reported that dead bacteria, compared with live bacteria, are enriched in cyclic adenosine monophosphate (cAMP). This observation raises the possibility that bacterial-derived nucleotide can contribute to the intracellular AMP pool and promote activation of the AMPK pathway [68]. Although this mechanism could potentially explain the AMPK phosphorylation observed with the heat-treated blend, it remains speculative and requires dedicated investigation. Importantly, while AMPK activation was detected, the absence of measurements of downstream effectors such as PGC-1α, NRF2, and their target proteins limits mechanistic interpretation. Therefore, additional studies are valuable to establish whether the AMPK/PGC-1α/NRF2 axis is responsible for the observed biological responses.

Additionally, intense exercise has been related to increase gastrointestinal permeability [69,70]. Inflammatory and pro-oxidative stimulus play a key role in this process, contributing to the destruction of the epithelial barrier and the severity of inflammation [71,72,73]. In the present study, the heat-treated blend decreased the IL-8 release in HT-29 enterocytes under a pro-inflammatory stimulus to aa similar extent as the probiotic blend. Accordingly, an improvement in intestinal barrier permeability was observed in both Caco-2 cell culture and a *C. elegans* model treated with the blends and exposed to pro-oxidative conditions. In line with our results, other studies have observed that postbiotics in the form of EPS or lipoteichoic acid (LTA) from bacterial species [51,74,75,76], heat-treated strains [31], or a blend of extracted glucans and inactivated yeasts [77] maintained intestinal barrier function through anti-inflammatory activities in other preclinical approaches.

Moreover, some probiotics and/or postbiotics have been reported to enhance the innate immune response by stimulating macrophages and dendritic cells located in the lamina propria [78,79]. This immunoregulatory effect may be mediated by microbial structural components, which can remain biologically active even after bacterial inactivation, thereby contributing to the immunomodulatory properties of postbiotic preparations [80,81,82]. In this study, both the probiotic and the heat-treated blend displayed immunomodulatory activity in U937-derived macrophages at the concentrations evaluated. Both blends stimulated the production of pro-inflammatory cytokines, as well as regulatory and anti-inflammatory mediators, suggesting a mixed activation profile rather than a strictly polarized macrophage response. Such a balanced cytokine pattern may be beneficial for maintaining immune homeostasis, by enhancing innate immune responsiveness while limiting excessive or prolonged inflammatory reactions. The increase in IL-6 and TNF-α release in the macrophages induced by the blends in both forms is commonly associated with the M1 macrophage phenotype. This activated form of macrophage is generally associated with an increase in phagocytic capacity and defense against bacterial and viral infections [83]. M1 macrophages act as the first line of defense against intracellular pathogens through different mechanisms of action. These include the production of ROS, the promotion of antigen presentation, endocytosis, and the induction of a T helper 1 shift of CD4+ T cells, enhancing the response against infections [84]. Although extrapolation from in vitro findings should be made with caution, these effects may be relevant in the context of athletic performance, where periods of intense training can transiently impair immune function. In this sense, previous studies have observed reductions in the prevalence or duration of infectious diseases in high-performance athletes after consumption of probiotics [85,86,87], even in a heat-treated form [88], possibly related to their immunomodulatory potential. However, the clinical relevance of this immunomodulatory effect remains controversial, since there are some studies that, despite observing immunomodulatory activity, did not observe a reduction in the number, duration or symptomatology of infectious diseases in high-performance athletes after the consumption of probiotics [42,85,89]. Furthermore, the stimulation of G-CSF secretion in U937 macrophages suggests activation of pathways involved in neutrophil regulation. Nevertheless, the physiological implications of this response remain uncertain. While G-CSF-mediated neutrophil mobilization may support innate immune defense and tissue repair processes, excessive neutrophil activity has also been associated with increased inflammatory burden and oxidative tissue damage. Therefore, the present findings suggest that both probiotic and heat-treated blends promote macrophage activation while simultaneously inducing regulatory immune mediators, resulting in a balanced immunomodulatory response. Nevertheless, the temporal dynamics of pro-inflammatory and regulatory responses in macrophages upon exposure to the blends would be of particular interest and warrant further investigation to better characterize the immunomodulatory properties of both formulations.

Altogether, based on our data, we speculate that the heat-treated blend may represent a promising emerging postbiotic for incorporation into functional foods. The observed effects on oxidative stress, inflammation, immune-related responses and intestinal barrier function warrant further investigation in physiological and exercise-relevant models, as these are areas of growing interest within sports nutrition. The heat-treated blend may be incorporated into a broader range of applications, including complex food matrices such as beverages and carbonated drinks [90,91,92], which represents a meaningful advantage for the food and sports nutrition industries. However, future randomized, placebo-controlled sports nutrition-related clinical trials are needed to corroborate these effects in humans.

## 5. Conclusions

In conclusion, the results indicate that both the probiotic and heat-treated Active Lifestyle blends exhibit antioxidant effects by reducing ROS and increasing survival of *C. elegans* under conditions of oxidative stress, while reducing induced gut permeability and inflammation and exhibiting immunomodulatory activity. Additionally, the heat-treated blend increased AMPK phosphorylation, mitochondrial content and ATP levels in C2C12 myotubes, suggesting a potential effect on pathways involved in cellular energy metabolism.

Importantly, at the tested concentrations, the biological activity observed after heat treatment expands the potential applicability of the blend beyond conventional probiotic formats. Therefore, the beneficial health effects exerted in both in vitro and in vivo models provide a strong rationale for its use as a promising ingredient in sports nutrition and for further evaluation of its effects in a sports nutrition clinical study.

## Figures and Tables

**Table 1:** Immunomodulatory properties of the probiotic and heat-treated blends. Percentage of cytokine release compared to the positive control (LPS 5 µg/mL) by U937 macrophages incubated with the probiotic or the heat-treated blends (n = 3 independent experiments).

% vs. PC	NC	Blend	Blend HT
TNF-α	0.09 ± 0.05 ^B^	105.8 ± 78.2 ^A^	71.7 ± 35.0 ^A^
IL-1β	20.61 ± 18.3 ^B^	220.3 ± 40.7 ^A^	230.2 ± 143.9 ^A^
IL-18	65.24 ± 13.5 ^B^	117.0 ± 27.3 ^A^	142.0 ± 27.3 ^A^
IL-6	0.19 ± 0.18 ^B^	90.0 ± 79.7 ^A^	81.08 ± 28.5 ^A^
IL-10	4.52 ± 5.26 ^B^	86.2 ± 80.3 ^A^	68.0 ± 44.3 ^A^
IL-RA	16.63 ± 11.9 ^B^	130.2 ± 107.6 ^A^	99.7 ± 60.4 ^A^
G-CSF	39.0 ± 35.4 ^B^	192.9 ± 57.9 ^A^	152.8 ± 73.4 ^A^
GM-CSF	3.1 ± 4.2 ^B^	141.4 ± 58.2 ^A^	129.5 ± 29.5 ^A^
EGF	9.76 ± 3.9 ^B^	97.9 ± 30.9 ^A^	95.0 ± 27.5 ^A^

Data are represented as the mean ± SD. Different letters within a row indicate statistically significant differences (*p* < 0.05) based on Kruskal–Wallis with Dunn’s multiple comparisons test (TNF-α, IL-1β, IL-6, G-CSF, GM-CSF, IL-10, IL-1RA) and one-way ANOVA followed by Tukeys’s multiple comparisons test (IL-18, EGF). EGF: epidermal growth factor; G-CSF: granulocyte colony-stimulating factor; GM-CSF: granulocyte-macrophage colony-stimulating factor; IL-10: Interleukin 10; IL-1β: Interleukin 1β; IL-18: Interleukin 18; IL-6: Interleukin 6; IL-RA: Interleukin 1 Receptor Antagonist; LPS: lipopolysaccharide; NC: negative control; TNF-α: tumor necrosis factor α.

## Data Availability

The original contributions presented in this study are included in the article. Further inquiries can be directed to the corresponding author.
